# Investigating Social Media to Evaluate Emergency Medicine Physicians’ Emotional Well-being During COVID-19

**DOI:** 10.1001/jamanetworkopen.2023.12708

**Published:** 2023-05-10

**Authors:** Anish K. Agarwal, Juhi Mittal, Annie Tran, Raina Merchant, Sharath Chandra Guntuku

**Affiliations:** 1 Penn Medicine Center for Digital Health, Philadelphia, Pennsylvania; 2 Department of Emergency Medicine, University of Pennsylvania, Philadelphia; 3 Perelman School of Medicine, University of Pennsylvania, Philadelphia; 4 Computer and Information Science, University of Pennsylvania, Philadelphia; 5 Leonard Davis Institute of Health Economics, University of Pennsylvania, Philadelphia

## Abstract

**Importance:**

Emergency medicine (EM) physicians experience tremendous emotional health strain, which has been exacerbated during COVID-19, and many have taken to social media to express themselves.

**Objective:**

To analyze social media content from academic EM physicians and resident physicians to investigate changes in content and language as indicators of their emotional well-being.

**Design, Setting, and Participants:**

This cross-sectional study used machine learning and natural language processing of Twitter posts from self-described academic EM physicians and resident physicians between March 2018 and March 2022. Participants included academic EM physicians and resident physicians with publicly accessible posts (at least 300 total words across the posts) from the US counties with the top 10 COVID-19 case burdens. Data analysis was performed from June to September 2022.

**Exposure:**

Being an EM physician or resident physician who posted on Twitter.

**Main Outcomes and Measures:**

Social media content themes during the prepandemic period, during the pandemic, and across the phases of the pandemic were analyzed. Psychological constructs evaluated included anxiety, anger, depression, and loneliness. Positive and negative language sentiment within posts was measured.

**Results:**

This study identified 471 physicians with a total of 198 867 posts (mean [SD], 11 403 [18 998] words across posts; median [IQR], 3445 [1100-11 591] words across posts). The top 5 prepandemic themes included free open-access medical education (Cohen *d*, 0.44; 95% CI, 0.38-0.50), residency education (Cohen *d*, 0.43; 95% CI, 0.37-0.49), gun violence (Cohen *d*, 0.37; 95% CI, 0.32-0.44), quality improvement in health care (Cohen *d*, 0.33; 95% CI, 0.27-0.39), and professional resident associations (Cohen *d*, 0.33; 95% CI, 0.27-0.39). During the pandemic, themes were significantly related to healthy behaviors during COVID-19 (Cohen *d*, 0.83; 95% CI, 0.77-0.90), pandemic response (Cohen *d*, 0.71; 95% CI, 0.65-0.77), vaccines and vaccination (Cohen *d*, 0.60; 95% CI, 0.53-0.66), unstable housing and homelessness (Cohen *d*, 0.40; 95% CI, 0.34-0.47), and emotional support for others (Cohen *d*, 0.40; 95% CI, 0.34-0.46). Across the phases of the pandemic, thematic content within social media posts changed significantly. Compared with the prepandemic period, there was significantly less positive, and concordantly more negative, language used during COVID-19. Estimates of loneliness, anxiety, anger, and depression also increased significantly during COVID-19.

**Conclusions and Relevance:**

In this cross-sectional study, key thematic shifts and increases in language related to anxiety, anger, depression, and loneliness were identified in the content posted on social media by academic EM physicians and resident physicians during the pandemic. Social media may provide a real-time and evolving landscape to evaluate thematic content and linguistics related to emotions and sentiment for health care workers.

## Key Points

**Question:**

How have emergency medicine physicians’ social media content and language changed during COVID-19, and can they provide insight into physician well-being?

**Findings:**

In this cross-sectional study of 471 academic emergency physicians and resident physicians, key differences in the social media content of before and during the pandemic were identified, including language associated with higher levels of anxiety, anger, and depression during the pandemic.

**Meaning:**

These findings suggest that social media content can provide insight into emergency medicine physicians’ mental well-being and may reveal signals related to burnout by identifying higher levels of mental health strain and key changes in thematic content.

## Teaser

This cross-sectional study analyzes social media content from academic emergency medicine physicians and resident physicians to investigate changes in content and language, from before vs during the COVID-19 pandemic, as indicators of their emotional well-being.

## Introduction

The COVID-19 pandemic continues to exacerbate mental health symptoms and health care–associated burnout across the clinical workforce.^1,2,3^ Burnout in health care and symptoms of depression and anxiety existed before the pandemic and across medical trainees (eg, students and resident physicians).^4,5^ COVID-19 continues to impact emergency medicine (EM) physicians and resident physicians.^6,7^ The frontline nature of providing care in the emergency department (ED) is intertwined with factors associated with stress, anxiety, depression, and burnout because of the chaotic environment, high rates of workplace violence, and constant exposure to critical care events.^6,8,9^

The initial and subsequent surges throughout the COVID-19 pandemic elevated public attention to burnout in health care and its impact on the mental health of the workforce. The US Surgeon General and the National Academy of Medicine have released a comprehensive report and call to action to specifically address the health care workforce highlighting the need to understand and support physicians.^10,11^ Physicians have begun sharing their perspectives more openly, and yet there is substantial stigma associated with accessing mental health resources and care medicine.^12,13^ The initial uncertainties and fears associated with the early pandemic were heightened for EM physicians, given the frontline nature of acute care delivery.^7^ Understanding the unique experiences and attitudes and capturing the voices of EM physicians and residents is important to identify and build strategies to support the workforce and trainees.

The exponential increase in the use of social media continues throughout the US and includes a growing landscape of medical education, clinician perspectives, and research.^14^ Platforms provide a large, unique, and accessible data source to understand the organic content posted by users. Many studies have used machine learning (ML) and natural language processing techniques to evaluate social media post content to identify themes related to health care and health, including mental and emotional health.^15,16^

An opportunity has emerged to investigate the social media content of EM physicians and resident physicians, to explore new data sources, to better identify emotional health strain, and to develop in-time interventions. The objective of this study was to use natural language processing and ML to evaluate Twitter posts by EM physicians throughout the pandemic, to identify themes in content and language related to psychological and mental health constructs.

## Methods

### Study Design and Setting

This cross-sectional study was designed to identify changes in psychological constructs from language posted to Twitter throughout the course of the pandemic from social media posts of EM physicians and resident physicians. This study was considered exempt from review and the need for informed consent by the University of Pennsylvania institutional review board, because the social media posts were publicly accessible. Specifically, this study sought to investigate how EM physicians’ and resident physicians’ social media language has changed during COVID-19, with particular attention to mental health. To answer these questions, this cross-sectional study analyzed publicly available social media content posted by EM physicians over a 4-year period between March 2018 and March 2022. The study followed the Strengthening the Reporting of Observational Studies in Epidemiology (STROBE) reporting guidelines for cross-sectional studies.^17^

### Selection of Participants and Outcomes

To optimize the ability to identify potential signals between COVID-19 case burden and language content on social media, the study focused on the top 10 counties in the US with the largest COVID-19 case counts at the time of the analysis using national data sets,^18,19^ which share cumulative COVID-19 cases by county. The top 10 counties included in the analysis were Los Angeles, California; Maricopa, Arizona; New York, New York; Miami-Dade, Florida; Cook, Illinois; Harris, Texas; San Diego, California; Riverside, California; Broward, Florida; and San Bernardino, California.

Next, EDs and training programs were matched to the aforementioned counties to determine those located in those counties using web-based mapping software (eg, Google Maps). We then searched the health system, hospital, and program’s website for the roster of physicians and resident physicians for each year. Twitter handles of EM physicians and resident physicians were then identified, and a data set was created for physicians, program, level of training, and social media (eg, handle). We verified profile photos, biographical information, and previous posts to validate that the handle corresponded to the physician resident. If we could not verify that a profile corresponded to a name listed, it was not included. We used the application programming interface to collect the most recent 3200 posts (Tweets) for each user posted between January 2018 and March 2022. This resulted in an initial sample of 199 368 posts from 569 users. We limited our analytic sample to users who wrote at least 300 words across their posts to obtain reliable language signal, on the basis of prior works.^20^ Outcomes included thematic content of posts, variation in content within posts over time, and linguistic attributes related to mental health within post content.

### Identifying Peaks and Troughs During COVID-19

The objective was to identify linguistic attributes in physician posts that significantly changed before and during COVID-19 and across the different phases of the pandemic. Posts before March 2020 are tagged as pre–COVID-19, and those from March 2020 onward are tagged as those posted during the pandemic. To identify different phases during COVID-19, we used the ruptures library in the Python programming language version 3.4 (Python Software Foundation)^21^ to identify the peaks and troughs in the cumulative COVID-19 cases of the counties under consideration for this study.

### Linguistic Attributes

We split posts into words, punctuation, and emoticons using the Happier Fun Tokenizer version 2017 (DLATK). To ensure that language markers identified in our analysis generalize outside of our sample, words and phrases used by less than 1% of users were excluded from the analysis.

We used the MALLET implementation of latent Dirichlet allocation version 2013 (Mimno) to identify latent data-driven word clusters (topics). We generated a set of 200 topics using all posts in our data set with α = .30 to favor fewer topics per document. We aggregated posts by month for each physician. An individual’s posts in each month were then represented by the probability of the physician mentioning each topic in the month, which is the combined probability of the physician mentioning a word and the probability that the words occur in the given topic. Every physician was then represented by the likelihood of mentioning each of the 200 topics per month. Latent Dirichlet allocation was used because it was shown in prior work^22^ to obtain qualitatively better topics compared with other topic-modeling approaches.

We used pretrained language models obtained from large-scale data sets to derive the standardized psychological constructs of individuals from their language each month. Specifically, we obtained sentiment, loneliness,^23^ anxiety,^24^ anger, depression,^25^ and positive and negative emotions. These models have been validated both in-sample and out-of-the-sample across multiple populations before and during COVID-19.^15,16^

### Identifying Differentially Expressed Language Features per Phase

Language attributes obtained per user per month were used as input variables, and the periods before and during COVID-19 were converted into a dummy variable to be used as the outcome in an ordinary least squares model. For identifying language associated with each of the 4 phases during COVID-19, we encoded each phase as a dummy and ran a similar ordinary least squares model to compute significant themes and constructs in each of the 4 phases.

### Statistical Analysis

Effect sizes were measured by Cohen *d* on the basis of the normalized frequency of the features.^26^ We used Benjamini-Hochberg *P* correction to correct for multiple comparisons and used 2-sided *P* < .05 for indicating significant associations. Data analysis was performed from June to September 2022.

## Results

The cross-sectional study period was from January 1, 2018, to March 31, 2022, where the preperiod was defined as January 1, 2018, to February 28, 2020. The final data set of EM physicians and residents posting from the counties with the top 10 COVID-19 case burden, with at least 300 words in posts over the study period, resulted in a final set of 471 users with a total of 198 867 posts. Participants in our study wrote a mean (SD) of 11 403 (18 998) words across posts (median [IQR], 3445 [1100-11 591] words across posts) between January 2018 and March 2022. Data were accessed and analyzed as a cross-section over the study period, and no participants were lost to follow-up.

We then computationally obtained 4 phases according to cumulative COVID-19 cases between March 1, 2020, to March 31, 2022. Phase 1 was from March 1, 2020, to October 31, 2020; phase 2 was from November 1, 2020, to April 30, 2021; phase 3 was from May 1, 2021, to December 31, 2021; and phase 4 was from January 1, 2022, to March 31, 2022. The number of EM physicians posting actively changed from 405 physicians (67 685 posts; mean [SD], 167.12 [311.04] posts per physician) before COVID-19 to 536 physicians (131 683 posts; mean [SD], 245.67 [494.97] posts per physician) during the pandemic.

### Changes in Social Media Content Across the Pandemic

The top 5 themes significantly associated with posts by EM physicians before March 2020 were about free open-access medical education in EM (Cohen *d*, 0.44; 95% CI, 0.38-0.50), residency education generally (Cohen *d*, 0.43; 95% CI, 0.37-0.49), gun violence (Cohen *d*, 0.37; 95% CI, 0.32-0.44), quality improvement in health care (Cohen *d*, 0.33; 95% CI, 0.27-0.39), and resident professional societies (Cohen *d*, 0.33; 95% CI, 0.27-0.39). The top 5 themes during the pandemic were significantly different and included healthy behaviors during COVID-19 (Cohen *d*, 0.83; 95% CI, 0.77-0.90), pandemic response (Cohen *d*, 0.71; 95% CI, 0.65-0.77), vaccines and vaccination (Cohen *d*, 0.60; 95% CI, 0.53-0.66), unstable housing and homelessness (Cohen *d*, 0.40; 95% CI, 0.34-0.47), and emotional support for others (Cohen *d*, 0.40; 95% CI, 0.34-0.46). Table 1 shows the top 5 significant topics before and during COVID-19 along with the associated effect sizes.^26^

**Table 1.** Themes Within Social Media Posts Before and During the COVID-19 Pandemic

Phase and topic	Top words and hashtags	Cohen *d* (95% CI)^a^
Before COVID-19 (January 1, 2018, to February 28, 2020)		
Free open-access medical education	#foamed, #meded, podcast, #foamped, check, roland, educator, #emconf, #emergencymedicine	0.44 (0.38-0.50)
Residency education	dr, residents, em, resident, conference, sim, teaching, #meded, learning, #whynuem	0.43 (0.37-0.49)
Gun violence	trauma, violence, gun, injuries, mass, children, injury, prevention, guns, police	0.37 (0.32-0.44)
Quality improvement in health care	data, tech, health care, #ehr, humanitarian, future, using, improve, clinical, systems	0.33 (0.27-0.39)
Professional resident associations	em, awesome, excited, emra, come, #emraleaders, #emraatacep18, meeting, conference, ready	0.33 (0.27-0.39)
During COVID-19 (March 1, 2020, to March 31, 2022)		
Healthy behaviors during COVID-19	covid, masks, #covid19, mask, testing, home, sick, wear, risk, protect	0.83 (0.77-0.90)
Pandemic response	pandemic, #covid19, public, health care, during, response, crisis, workers, country, covid-19	0.71 (0.65-0.77)
Vaccines and vaccination	vaccine, covid, vaccinated, vaccines, world, shot, #covid19, getting, flu, vaccination	0.60 (0.53-0.66)
Homelessness and unstable housing	cases, nyc, covid, homelessness, homeless, housing, shelters, #covid19, shelter, deaths	0.40 (0.34-0.47)
Emotional support for others	sorry, hope, well, re, hear, glad, soon, am, sending, doing	0.40 (0.34-0.46)

^a^ All topics are significant at *P* < .01 after Benjamini-Hochberg multiple hypothesis correction.

Across the phases of the pandemic, thematic content within social media posts also changed significantly. Within phase 1, the top 5 themes were COVID-19 (Cohen *d*, 0.51; 95% CI, 0.44-0.59), public health safety (Cohen *d*, 0.43; 95% CI, 0.35-0.51), mask wearing (Cohen *d*, 0.28; 95% CI, 0.20-0.35), racial equity (Cohen *d*, 0.24; 95% CI, 0.17-0.32), and stay-at-home measures (Cohen *d*, 0.23; 95% CI, 0.16-0.31). Phase 2 themes shifted to vaccination, hope, political discourse, medical students and residents, and condolences to others. Table 2 shows the top themes in social media posts and associated effect sizes.

**Table 2.** Themes Across Phases During the Pandemic

Phase and topic	Top words and hashtags	Cohen *d* (95% CI)^a^
Phase 1 (March 1 to October 31, 2020)		
COVID-19 general	pandemic, #covid19, public, health care, during, response, crisis, workers, country, covid-19	0.51 (0.44-0.59)
Public health safety	covid, masks, #covid19, mask, testing, home, sick, wear, risk, protect	0.43 (0.35-0.51)
Mask wearing	wear, wearing, hair, mask, off, face, down, cut, scrubs, eye	0.28 (0.20-0.35)
Race and equity	black, racism, white, against, police, must, justice, social, race, racial	0.24 (0.17-0.32)
Stay at home	keep, stay, safe, going, mind, home, please, keeping, open, coming	0.23 (0.16-0.31)
Phase 2 (November 1, 2020, to April 30, 2021)		
Vaccination	vaccine, covid, vaccinated, vaccines, world, shot, #covid19, getting, flu, vaccination	0.36 (0.28-0.43)
Hope	happy, family, welcome, birthday, year, class, excited, wait, christmas, everyone	0.30 (0.23-0.38)
Political discourse	vote, president, trump, news, america, country, election, states, obama, debate	0.20 (0.13-0.28)
Medical students and residency match	em, #medtwitter, interview, program, residency, programs, #embound, match, students, #medstudenttwitter	0.17 (0.09-0.24)
Condolences	sorry, hope, well, re, hear, glad, soon, am, sending, doing	0.14 (0.06-0.21)
Phase 3 (May 1 to December 31, 2021)		
Vaccination	vaccine, covid, vaccinated, vaccines, world, shot, #covid19, getting, flu, vaccination	0.32 (0.24-0.40)
Teamwork	game, win, play, team, world, player, players, football, fan, big	0.19 (0.11-0.26)
Excitement in future	research, space, excited, program, team, learn, leadership, project, community, group	0.17 (0.09-0.25)
Congratulations	congrats, congratulations, dr, well, proud, amazing, deserved, friend, award, mentor	0.17 (0.09-0.24)
Opioid epidemic	use, opioid, drug, drugs, overdose, treatment, naloxone, opioids, epidemic, via	0.16 (0.08-0.23)

^a^ All topics are significant at *P* < .01 after Benjamini-Hochberg multiple hypothesis correction. There were no significant topics in phase 4 (January 1 to March 31, 2022).

### Changes in Mental Well-being and Emotions Across the Pandemic

**Figure 1.**
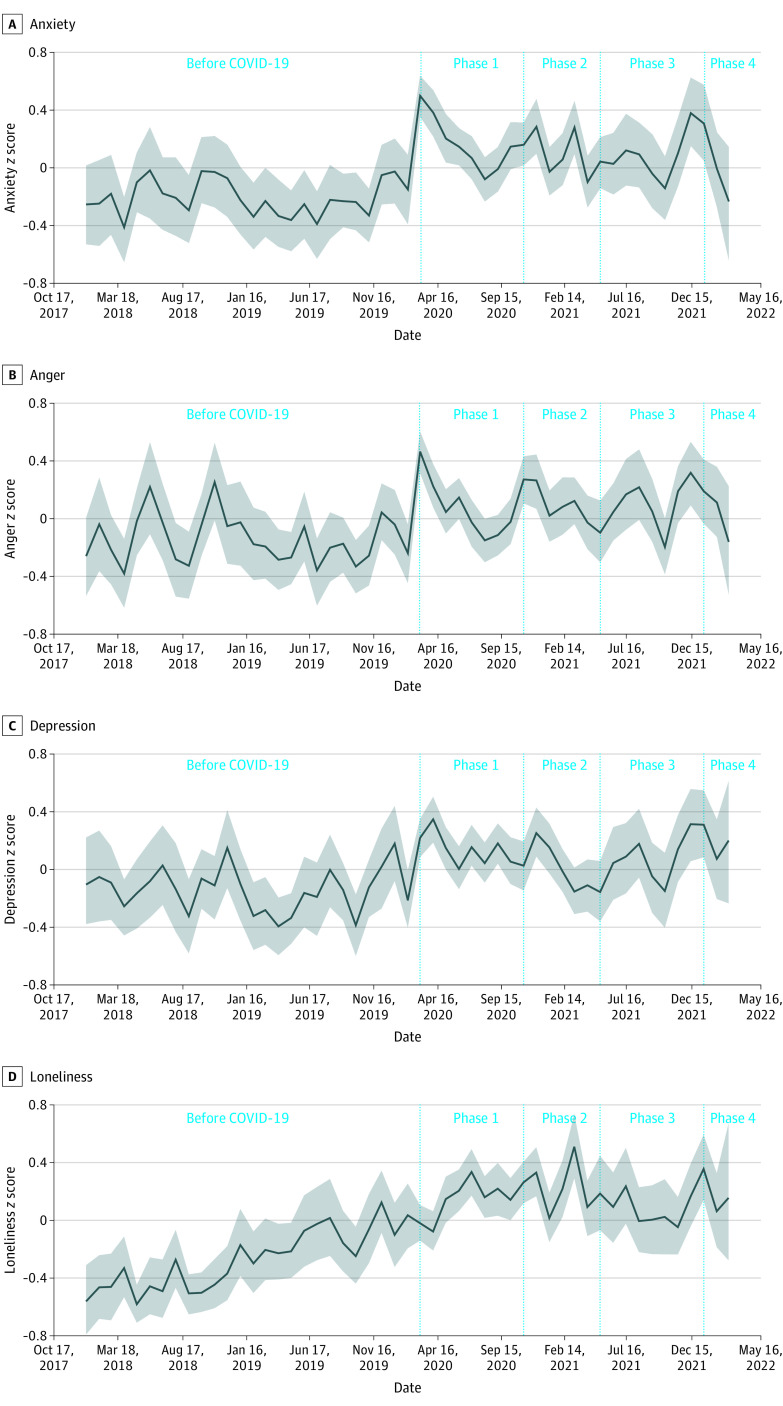
Psychological Constructs Across Phases of the COVID-19 Pandemic Graphs show *z* scores for anxiety (A), anger (B), depression (C), and loneliness (D). Solid lines denote mean values, and shaded areas denote 95% CIs.

**Figure 2.**
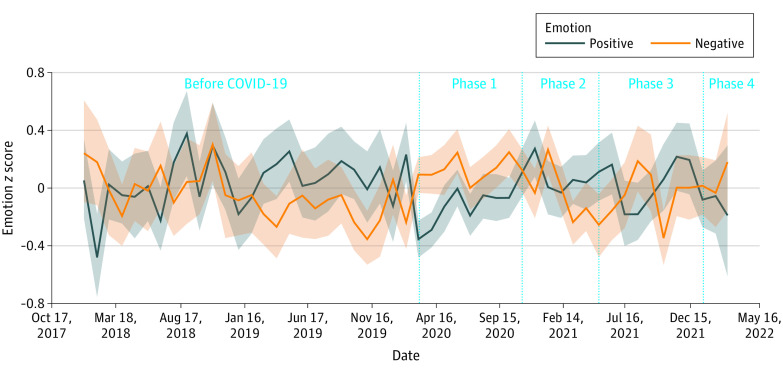
Positive and Negative Language Sentiment Across Phases of the COVID-19 Pandemic Graph shows *z* scores for positive and negative emotions over time. Solid lines denote mean values, and shaded areas denote 95% CIs.

Compared with the prepandemic period, there was significantly less positive, and concordantly more negative, language used during COVID-19. Estimates of loneliness, anxiety, anger, and depression also increased significantly during COVID-19. Figure 1 shows changes across these psychological language constructs over time, and Figure 2 shows effect sizes of positive and negative language over time. Most specifically, the largest changes were seen between the pre–COVID-19 period and phase 1 with significant decreases in positive language use and significant increases in negative language use, loneliness, anxiety, anger, stress, and depression. Across phases there was variation across these constructs (eFigure in Supplement 1).

## Discussion

COVID-19 has placed an extraordinary amount of physical and psychological stress on health care systems and the health care workforce.^3,27^ This is especially true for frontline clinical specialties such as EM and for trainees such as resident physicians.^2,6,7,8^ This cross-sectional study analyzed social media posts from EM physicians and resident physicians from counties with high COVID-19 case burdens to evaluate change in linguistic content and emotion using ML and natural language processing.

This study has 3 significant findings. First, self-identified EM physicians and resident physicians were posting large amounts of content throughout the periods before and during the COVID-19 pandemic. As for other populations, Twitter serves as a large source for this content and a proxy for the topics and language used related to mental health.^16,20,28,29^ Social media platforms provide unique and dynamic environments for users to share their personal opinions and thoughts and spaces to collect data at scale. We were able to identify physicians and resident physicians on Twitter using traditional methods. What remains unknown is the content posted by users who designed their profiles to be less identifiable (eg, profile not easily linked to individual by username or photo) and how variations in social media platform popularity will change use in the wake of new emerging platforms (TikTok), changes in ownership over large platforms (Twitter purchase^30^), and overall social media use moving forward.^31^ Over the course of this study, we saw consistent use without large variation in posting.

Second, this study reveals significant spikes in negative psychological constructs within Twitter language across EM physicians. In this study, we identified significantly higher psychological strain across all domains and higher negative language across the pandemic. Leading into the pandemic, we saw a general increase in language expressing loneliness in the content, in line with the growing literature on the presence of loneliness.^28,32,33,34^ The focus on loneliness is also interesting, given its increasing connection to burnout among health care workers. Across constructs, we saw striking shifts throughout the pandemic that could be identified through their social media content. These signals provide another method of evaluating the mental health of the workforce and those in training at scale. These findings provide insights into the mental health of physicians using new methods to understand psychological and emotional health via social media. To our knowledge, this study is among the first to deploy ML to analyze this content at scale for the health care population and to understand another dynamic aspect of their mental and emotional health. Large survey-based methods have also identified these trends,^35^ yet social media provides an organic and unstructured environment to investigate thematic content and emotion in the moment.

Third, this study identified key topics that physicians and resident physicians were discussing on social media throughout phases of the pandemic. These topics varied and provided a novel approach to understanding what topics were prescient to residents in training. In our analyses, we found trends that matched national and social topics regarding injustice, including gun violence, the opioid epidemic, racism, and equity. This certainly may be unique to EM given its intersectionality with public health and health care; however, we hypothesize that these topic areas are cutting across to other specialty areas. Future work can analyze the variation in topic content across training fields (eg, surgery, medicine, and pediatrics) and across geographic regions. In addition, the landscape of social media platforms continues to change with variation in user preference and popularity. Similar large ML approaches could be applied to alternative platforms to identify and validate themes within varying content types, including written language and video-based and photo-based posts.

### Limitations

This study has limitations that are important to note. It uses social media content, which is biased by those who are actively posting on social media; thus, participants who may be more inclined to share their opinions, emotions, or thoughts may be overrepresented. This analysis used data from the Twitter platform and did not aggregate data from other social media platforms (eg, Instagram, TikTok, or Facebook) and, thus, the findings may not be generalizable to all social media content and is limited to those users on Twitter. Our approach included publicly or easily identifiable EM physicians and residents who either shared their social media usernames or accounts or those who identified themselves on the platform. This analysis would have otherwise not included individuals with a username or account that is not identifiable or attributable to the individuals; furthermore, individuals may have multiple accounts. In this analysis, we chose to only analyze individuals in counties with the top 10 highest COVID-19 case burden, which introduces selection bias. The purpose of this selection was to determine whether a signal was present and to look within potentially high-risk or high-exposure populations. This strategy is nonetheless limited because it does not include individuals outside those areas and may represent skewed results. Future work can look more broadly across the nation and in varying fields in medicine. This approach did not account for the volume of posts and is correlational, not causal.

## Conclusions

Social media can provide a rich, dynamic, and unique window into the emotions, thoughts, and opinions of individuals. As those in health care continue to face the multiple mental health strains associated with providing care, exacerbated by the COVID-19 pandemic, they may share their thoughts and emotions on social media platforms. This cross-sectional study reveals a significant increase in mental health strain among EM physicians and residents at the onset and persistently throughout the pandemic. It also highlights key topic areas that leaders and educators can use to inform their approaches to supporting the rising workforce during their training.

## Notes

Supplement 1. **eFigure.** Change in Language Constructs Across Phases (T-Test Output)

Supplement 2.
Data Sharing Statement
