# Supplementary material for: Investigating Social Media to Evaluate Emergency Medicine Physicians’ Emotional Well-being During COVID-19
Source: JAMA Netw Open. 2023 May 10;6(5):e2312708. doi: 10.1001/jamanetworkopen.2023.12708 (PMC10173019; doi:10.1001/jamanetworkopen.2023.12708)
Supplement: Supplement 1. — eFigure. Change in Language Constructs Across Phases (T-Test Output) [file jamanetwopen-e2312708-s001.pdf]

Supplemental Online Content

Agarwal AK, Mittal J, Tran A, Merchant R, Guntuku SC. Investigating social media to evaluate emergency medicine physicians' emotional well-being during COVID-19. *JAMA Netw Open*. 2023;6(5):e2312708. doi:10.1001/jamanetworkopen.2023.12708

eFigure. Change in Language Constructs Across Phases (T-Test Output)

This supplemental material has been provided by the authors to give readers additional information about their work.

© 2023 Agarwal AK et al. *JAMA Network Open.*

eFigure. Change in Language Constructs Across Phases (T-Test Output)

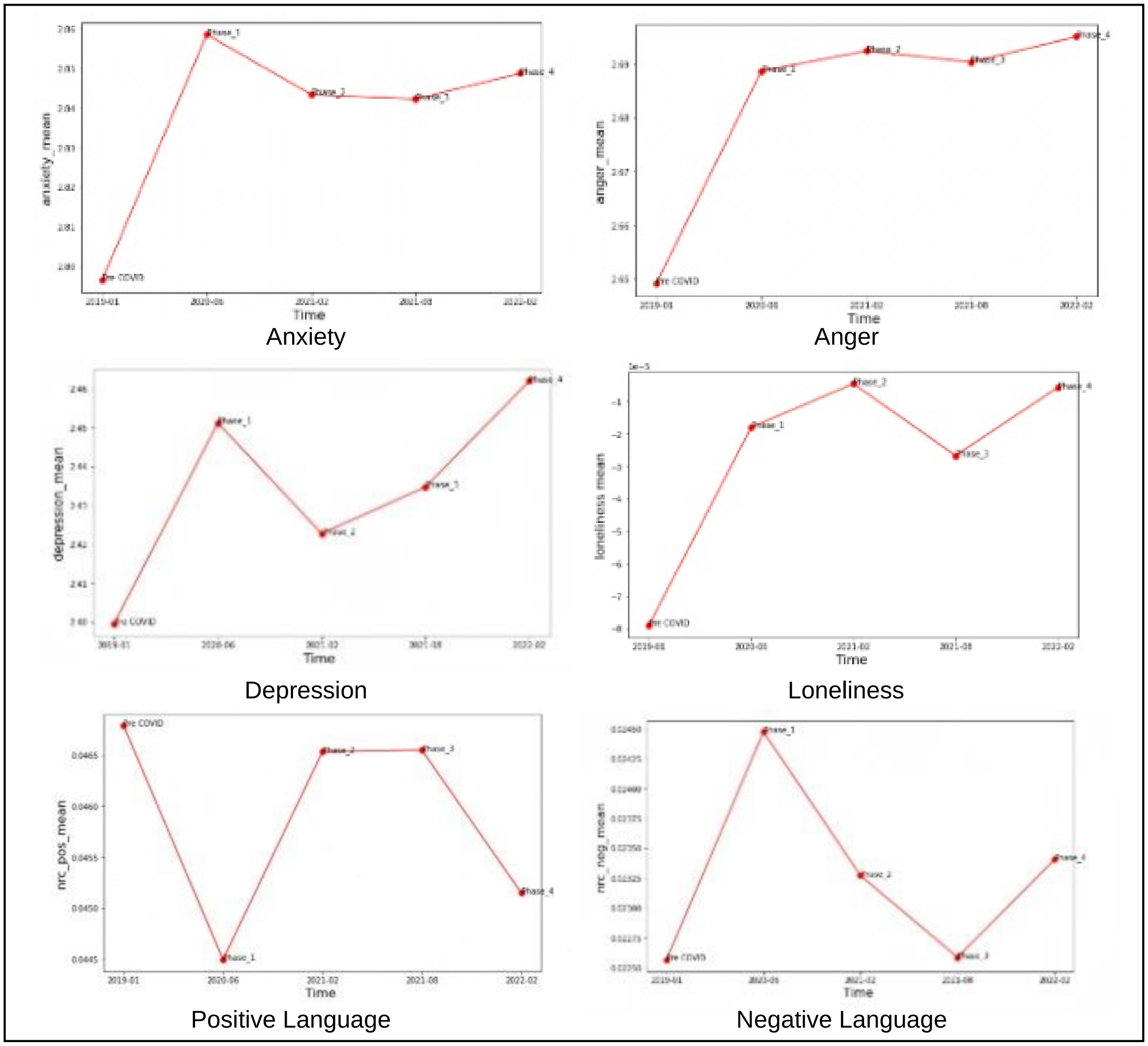

© 2023 Agarwal AK et al. *JAMA Network Open.*
