# Supplementary material for: Investigating Social Media to Evaluate Emergency Medicine Physicians’ Emotional Well-being During COVID-19
Source: JAMA Netw Open. 2023 May 10;6(5):e2312708. doi: 10.1001/jamanetworkopen.2023.12708 (PMC10173019; doi:10.1001/jamanetworkopen.2023.12708)
Supplement: Supplement 2. — Data Sharing Statement [file jamanetwopen-e2312708-s002.pdf]

Data Sharing Statement

Agarwal. Investigating Social Media to Evaluate Emergency Medicine Physicians' Emotional Well-being During COVID-19. *JAMA Netw Open*. Published May 10, 2023.
doi:10.1001/jamanetworkopen.2023.12708

Data
Data available: Yes
Data types: Other (please specify)
Additional Information: Social Media Data
How to access data: anish.agarwal@pennmedicine.upenn.edu
When available: With publication

Supporting Documents
Document types: Informed consent form
How to access documents: anish.agarwal@pennmedicine.upenn.edu
When available: With publication

Additional Information
Who can access the data: researchers whose proposed use of the data has been approved
Types of analyses: for any purpose or for a specified purpose
Mechanisms of data availability: with investigator support, after approval of a proposal, or with a signed data access agreement
